# Label-free impedimetric immunosensor for point-of-care detection of COVID-19 antibodies

**DOI:** 10.1038/s41378-022-00460-5

**Published:** 2023-01-01

**Authors:** Lian C. T. Shoute, Gaser N. Abdelrasoul, Yuhao Ma, Pedro A. Duarte, Cole Edwards, Ran Zhuo, Jie Zeng, Yiwei Feng, Carmen L. Charlton, Jamil N. Kanji, Shawn Babiuk, Jie Chen

**Affiliations:** 1 Department of Electrical and Computer Engineering, University of Alberta, Edmonton, AB T6G 2V4 Canada; 2 Public Health Laboratory, Alberta Precision Laboratories, Edmonton, AB Canada; 3 Department of Laboratory Medicine and Pathology, University of Alberta, Edmonton, AB T6G 2B7 Canada; 4 Li Ka Shing Institute for Virology, University of Alberta, Edmonton, AB Canada; 5 Division of Infectious Diseases, Department of Medicine, Cumming School of Medicine, University of Calgary, Calgary, AB Canada; 6 Department of Pathology & Laboratory Medicine, Cumming School of Medicine, University of Calgary, Calgary, AB Canada; 7 National Centre for Foreign Animal Disease, Canadian Food Inspection Agency, Winnipeg, MB Canada; 8 Department of Immunology, University of Manitoba, Winnipeg, MB Canada; 9 Department of Biomedical Engineering, University of Alberta, Edmonton, AB T6G 2R3 Canada

**Keywords:** NEMS, Biosensors

## Abstract

The COVID-19 pandemic has posed enormous challenges for existing diagnostic tools to detect and monitor pathogens. Therefore, there is a need to develop point-of-care (POC) devices to perform fast, accurate, and accessible diagnostic methods to detect infections and monitor immune responses. Devices most amenable to miniaturization and suitable for POC applications are biosensors based on electrochemical detection. We have developed an impedimetric immunosensor based on an interdigitated microelectrode array (IMA) to detect and monitor SARS-CoV-2 antibodies in human serum. Conjugation chemistry was applied to functionalize and covalently immobilize the spike protein (S-protein) of SARS-CoV-2 on the surface of the IMA to serve as the recognition layer and specifically bind anti-spike antibodies. Antibodies bound to the S-proteins in the recognition layer result in an increase in capacitance and a consequent change in the impedance of the system. The impedimetric immunosensor is label-free and uses non-Faradaic impedance with low nonperturbing AC voltage for detection. The sensitivity of a capacitive immunosensor can be enhanced by simply tuning the ionic strength of the sample solution. The device exhibits an LOD of 0.4 BAU/ml, as determined from the standard curve using WHO IS for anti-SARS-CoV-2 immunoglobulins; this LOD is similar to the corresponding LODs reported for all validated and established commercial assays, which range from 0.41 to 4.81 BAU/ml. The proof-of-concept biosensor has been demonstrated to detect anti-spike antibodies in sera from patients infected with COVID-19 within 1 h.

**Figure:**
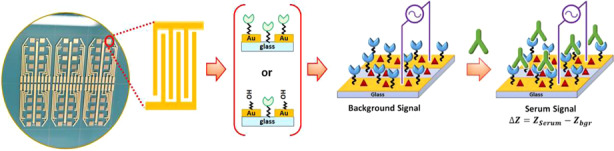
Photolithographically microfabricated interdigitated microelectrode array sensor chips & label-free impedimetric detection of COVID-19 antibody.

Photolithographically microfabricated interdigitated microelectrode array sensor chips & label-free impedimetric detection of COVID-19 antibody.

## Introduction

Coronavirus 2019 (COVID-19) is caused by the “severe acute respiratory syndrome coronavirus 2” virus (SARS-CoV-2), as designated by the International Committee on Taxonomy of Viruses^1–3^. Because the virus is highly contagious and can cause severe respiratory illness and death, the World Health Organization labeled the COVID-19 outbreak as a pandemic on March 11, 2020^4^. The size of SARS-CoV-2 virion ranges from 60–140 nm and spike-like projections of glycoproteins cover its surface^1,5^. SARS-CoV-2 is an enveloped positive-sense single-stranded RNA virus with a genome size of ~30,000 bases long^1,6^, and the virus encodes 29 different proteins, including 4 structural, 16 nonstructural, and 9 accessory proteins^7,8^. The structural proteins, including spike (S), envelope (E), and membrane (M) proteins, create the viral envelope, and the nucleocapsid (N) proteins package the RNA genome and serves as a template for viral transcription and replication.

In a pandemic, the availability of fast, accurate, and accessible diagnostic methods to detect infections and monitor the immune response is essential for treating, mitigating, controlling and managing the spread of the disease^9–15^. At present, the main laboratory diagnostic tools available to detect and monitor SARS-CoV-2 infection can be classified into the following main categories: tools that (1) identify viral RNA, (2) detect viral antigen, and (3) measure antibodies produced in humoral response to the viral infection. Real-time reverse transcriptase polymerase chain reaction (RT‒PCR), which detects viral RNA, is the gold standard for diagnosing active infections^16–18^. The technique exhibits high sensitivity and specificity, as the targeted genes are exponentially amplified for detection. However, the technique is susceptible to sample handling problems and viral mutations, which can result in false-positive and false-negative test results, respectively. Antigen tests are immunoassays that detect proteins present in the virus, such as the N and S proteins, to identify active infection^19–22^. Rapid antigen tests based on lateral flow assays have become synonymous with COVID-19 point-of-care (POC) diagnostics, as the tests are easy to use, inexpensive and can provide test results in 5–30 min^23,24^. However, compared to RT‒PCR, rapid antigen tests are less sensitive but are less prone to sampling problems, as proteins are much more stable than viral RNA.

Antibody or serology test methods are designed to detect the presence of antibodies against SARS-CoV-2 in the human body^9,25–28^. Since antibodies are produced by B cells of the immune system in response to viral infection, positive serological tests indicate past as well as current infections; in addition, these tests are complementary to viral RNA and viral antigen tests because they can help reduce the number of false-positive and negative tests. In fact, serology tests exhibit a few advantages over viral RNA and antigen tests, including a much longer detection window, more stable human antibodies compared to those of viral RNA, safer method of collecting blood rather than respiratory samples, more uniform distribution of antibodies in blood than virus in respiratory samples and no requirement of a biosafety level (BSL)-2 laboratory. In addition, antibody tests play important roles in diagnosing suspected cases that involve a negative viral RNA test or asymptomatic infection and in contact tracing, surveillance, virus origin tracing and epidemiological assessment at a population level; the tests also play a role in monitoring immune responses to assess the course, degree, and durability of immunity as well as identifying potential convalescent plasma donors, developing and evaluating therapeutic antibodies, and developing and evaluating vaccines.

SARS-CoV-2 infection leads to the production of IgM, IgA, and IgG immunoglobulins against the most immunogenic structural spike (S) and nucleocapsid (N) proteins of the virus^29–34^. IgM and IgA are binding proteins, and IgG is the neutralizing protein. The binding antibodies signal the presence of a pathogen in the body, while neutralizing antibodies block the entry of a pathogen into a cell and can act as a protection against reinfection. IgM is considered an indicator of early-stage infection, while IgG is an indicator of current or prior infection. The viruses and the antibodies produced due to infection can be detected in the body after incubation and seroconversion, respectively. Experimental determination of the seroconversion time and the temporal dynamics of the individual antibodies due to SARS-CoV-2 infection is complex and remains controversial^35–40^. The median seroconversion times determined for total antibody, IgA, IgM, and IgG are 9–11, 5 or 13, 8–14, and 8–14 days after symptom onset, respectively. The average time to reach the highest titer is ~2 weeks for total antibodies, 2–3 weeks for IgA, 2–3 weeks for IgM, and 3–4 weeks for IgG after symptom onset. The antibodies IgA and IgM can persist in the body for ~2 months, while IgG can persist for more than 3 months^9,41–48^.

Many serological diagnostic techniques have been developed to detect immunoglobulins IgA, IgM, and IgG in the blood against immunogenic proteins of SARS-CoV-2. The standard laboratory serological diagnostic techniques include enzyme-linked immunosorbent assays (ELISAs), chemiluminescence immunoassays (CLIAs), and neutralization assays^49–56^. Although these techniques are sensitive, specific, and reliable, the assays require specialized laboratories, instruments, trained technicians, and hours or even days to perform the analysis and hence are not applicable in resource-poor regions in the world. Due to the highly contagious nature of SARS-CoV-2 and the worldwide occurrence of COVID-19, there is an enormous demand for improving existing diagnostic methods as well as developing new diagnostic methods and POC devices for fast, low-cost, portable, user-friendly, and accurate on-site detection at the point-of-need of patient.

Electrochemical biosensors exhibit immense potential for POC applications, as they are the most amenable sensors to miniaturization^57–67^. Biosensors are essentially an integration of the following components: biorecognition elements, transducers, and signal processors. In an electrochemical biosensor, the transducer is essentially an electrode made of inert conducting material such as gold and electrical transduction signals, which are detected as current, potential, or impedance and are unaffected by the color and opacity of the sample. Rashed et al.^68^ reported label-free electrochemical detection of SARS-CoV-2 antibodies using a 16-well platform with integrated electrodes and electrochemical impedance-based sensing (EIS). The 16-well plates were coated with SARS-CoV-2 spike receptor-binding domain (RBD) protein, and the antibody CR3022 was detected in less than 5 min by the impedance change recorded immediately after a sample containing CR3022 was added. Ali et al.^69^ reported a 3D nanoprinted gold micropillar array as the working electrode on a glass substrate with a patterned gold film, which formed the base of the working, counter, and reference electrodes. The electrodes were integrated with a microfluidic device and used in a standard electrochemical cell. Specific viral antigens (S1 and RBD protein) were immobilized on the reduced-graphene-oxide nanoflakes that coated the gold micropillar array. The binding of the IgG antibodies to the S1 and RBD proteins was probed with Faradaic EIS using a ferrous/ferric redox couple. Antibodies against SARS-CoV-2 spike S1 and RBD proteins were detected within a few seconds using a smartphone-based user interface. Yakoh et al.^70^ reported a paper-based electrochemical biosensor that contained a working ePAD, counter ePAD, and closing ePAD folded into an electrochemical cell, and this sensor detected antibodies of the RBD SARS-CoV-2 spike protein in 30 min. Hashemi et al.^71^ reported an electrochemical biosensor based on a glassy carbon electrode coated with graphene oxide that was conjugated to a gold nanostar system, and this sensor was capable of detecting traces of monoclonal IgG antibody against the S1 protein of SARS-CoV-2 within 1 min.

These reports have demonstrated that biosensors based on electrochemical transduction are applicable to miniaturization, can be integrated into microfluidics lab-on-a-chip systems, and meet fast assay requirements for POC applications^68–71^. However, the reported immunosensors for COVID-19 antibody detection are based on square-wave voltammetry (SWV), differential pulse voltammetry (DPV), and Faradaic and capacitive EIS detection. Electrochemical detection based on SWV, DPV and Faradaic EIS requires the sample solution to be doped with a redox couple to enhance the detection signal; thus, the method is not strictly regarded as label-free detection. The label-free capacitive EIS reported by Rashed et al.^68^ used the transient impedance decay of the interfacial capacitor^57^, which was connected in series with the solution resistance for the detection. This method exhibits drawbacks, as it is very susceptible to external electronic disturbances. Moreover, the observed transient impedance jumped, and subsequent decay occurred due to the addition of the sample, indicating that the antibody-antigen reaction likely cannot reach equilibrium in that time, considering that a 30 min incubation was used for the ELISA test. Similar arguments can be made against all the reported assays, which claimed to have a faster detection time than the minimum time necessary for the system to attain equilibrium. Therefore, it is highly desirable to develop a label-free capacitive immunosensor that can overcome the drawbacks associated with transient and other redox reagent-based electrochemical biosensors that were reported recently for COVID-19 antibody detection. In this report, we present a label-free capacitive immunosensor based on an interdigitated microelectrode array (IMA), which uses a low sinusoidal excitation voltage and eliminates the need for applying high DC voltages and redox reagents. The microfabricated gold IMA has a thickness, width, gap, and number of digits of 60 nm, 4 μm, 2 μm, and 500, respectively, to enhance the signal-to-noise ratio and sensitivity, which eliminates the need for a counter electrode in the electrochemical biosensor. Furthermore, the sensitivity of the device can be significantly enhanced by simply tuning the ionic strength of the solution. We have demonstrated that the label-free capacitive biosensor can detect clinically relevant concentrations of SARS-CoV-2-specific antibodies against the S protein present in human serum samples within an hour.

## Results and discussion

### Surface functionalization and S-protein immobilization

**Fig. 1:**
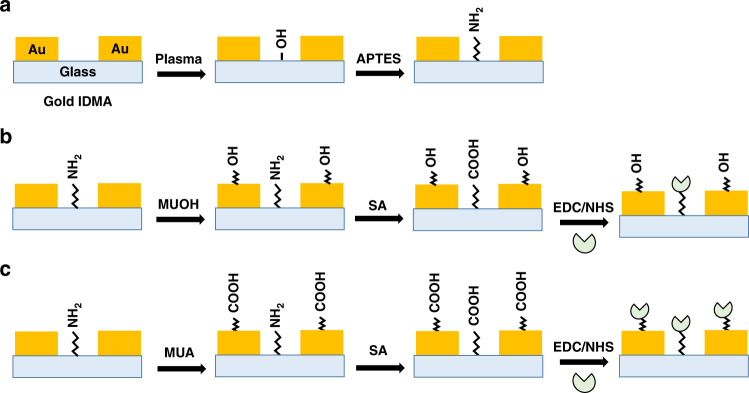
Schematic illustration of the surface functionalization of IMA and spike protein immobilization. **a** Hydroxyl groups produced on the glass surface upon plasma exposure react with APTES to form SAM with surface -NH_2_ groups, **b** MUOH forms SAM with surface -OH groups on the Au surface, and **c** MUA forms SAM with surface -COOH groups on the Au surface. The -NH_2_ group reacts with SA to functionalize the surface with -COOH groups in **b**, **c**, which on subsequent application of EDC/NHS conjugation chemistry led to covalent immobilization of the S-proteins ().

Surface functionalization and covalent immobilization of the SARS-CoV-2 spike proteins on the surface of the IMA are expected to provide enhanced stability and robustness to the device. These steps are also essential for the development of biosensors for sensitive and selective detection of low levels of target anti-S-protein antibodies that are present in the serum samples of COVID-19 patients. The antibody tests in this work used the S-protein of SARS-CoV-2. The S-protein is a homotrimer in which each monomer contains S1 and S2 subunits. The S1 subunit of the S-protein consists of the receptor-binding domain (RBD) and N-terminal domain (NDT), while the S2 subunit comprises the fusion peptide (FP), heptapeptide repeat HR1 and HR2, transmembrane (TM) and cytoplasm (CT) domains. The S1 and S2 subunits of the S-protein are responsible for recognizing and binding host cellular receptors, fusing the membrane and entering into host cells; thus, these subunits play a fundamental role in viral infection^29–34^. In addition, these units are highly immunogenic and hence are targets for vaccine development, antibody-blocking therapy, and small molecule inhibitors^29–34^. As presented schematically in Fig. 1a, hydroxyl groups formed on the glass surface of IMA upon plasma exposure readily react with APTES to form amine-terminated SAM, which on subsequent reaction with succinic anhydride led to SAM with surface carboxyl groups, as presented in Fig. 1b. To mitigate the potential effects caused by the nonspecific binding of proteins, which is due to passive adsorption on the surface of the bare electrodes and to facilitate sensitive detection using label-free impedimetric immunoassay, thiol chemistry was used to coat the gold IMA with an insulating layer of hydrophilic SAM layer that contains surface -OH groups formed by MUOH. Finally, standard EDC/NHS conjugation chemistry was applied to achieve covalent immobilization of the S-proteins on the surface of the IMA, as displayed in Fig. 1b. As seen in Fig. 1c, MUA was used instead of MUOH to investigate the effect of the SAM-coated Au surface with a distal -COOH group, which in turn can increase the amount of surface immobilized S-proteins, and the device performance.

### EIS characterization of functionalized surfaces

**Fig. 2:**
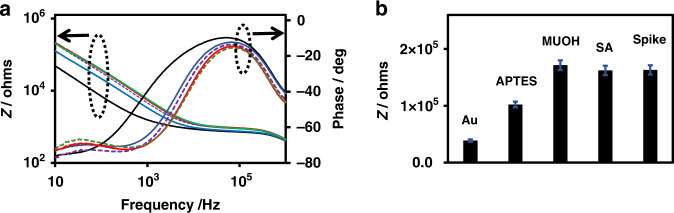
EIS characterization of the surface functionalization of IMA and spike protein immobilization. **a** Bode plot of bare gold (black line) IMA functionalized with APTES (blue line), MUOH (red line), SA (purple dashed line), and spike protein immobilization (green dashed line). **b** Plot of the impedance magnitude (*Z*) at 13.2 Hz recorded after each step of IMA functionalization.

The surface functionalization and S-protein immobilization of the IMA were characterized by non-Faradaic electrochemical impedance spectroscopy (EIS), which selectively probes the Au electrode-solution interface. Figure 2a shows the impedance spectra of IMA recorded after each step in a 1/1000 dilution of 1x PBS (0.001x PBS) at pH 7.4, which was achieved by applying a sinusoidal excitation voltage of ±10 mV at 0 V DC and scanning the frequency from 10 Hz to 1 MHz. Figure 2b displays the magnitude of the impedance at 13.2 Hz, which was determined after each step. The impedance magnitude value (*Z*) at a 13.2 Hz frequency was used in this work to determine the sensor response to the target analyte when it bound to the recognition element on the IMA transducer, as the impedance in the low frequency region is dominated by the interfacial capacitance of the system. In addition, this frequency is well within the optimum frequency range of 4–20 Hz that was empirically determined by the immittance approach for capacitive EIS immunosensors^72^. Interestingly, a dramatic change in the impedance spectrum, both in magnitude and phase, was observed upon APTES functionalization in the low frequency region of the spectrum, demonstrating that the functionalization has a strong effect on the interfacial capacitance. This effect can be rationalized as follows. It is reasonable to assume that the effect occurs because the applied electric field is strongly confined at the edges of the IMA electrodes. This proposition is supported by several recent reports on FEM and COMSOL simulations, which demonstrated that the applied electric field intensity in planar IDE electrodes is highly confined and is many orders of magnitude higher at the edges of the gold electrode^61,73,74^. High confinement of the electric field is expected for our planar gold IMA, as its width and thickness are 4 μm and 0.06 μm, respectively. Under this condition, partially coated gold edges could cause a dramatic increase in the impedance. Alternatively, the APTES coating of the gap region of IMA could alter the surface conductivity due to the formation of monolayer/multilayers of positively charged amine groups between the electrode digits. The formation of a highly charged layer of ammonium groups is expected to increase the gap conductivity, which is contrary to our observation. It is also reasonable to assume that an insulating coating formed by APTES in the gap region extends to coat the surface of the gold electrodes, which is due to the propensity of APTES to undergo extensive cross-linking reactions and polymer formation^61^. The proposed extensive coating of the gold electrode by APTES is contrary to the large increase in impedance observed as the SAM of MUOH formed on Au electrodes (Fig. 2b), indicating that the cross-linked APTES provides only partial coating to the Au electrode surface of the IMA. The large impedance increase observed upon APTES and MUOH functionalization can be attributed to the formation of insulation layers in SAM, which exhibit much smaller capacitances than the serially connected capacitance of the EDL in the system. After the IMA was coated with an insulating SAM layer of APTES and/or MUOH, any subsequent surface conjugation reactions were observed to have only a small and subtle effect on the impedance magnitude and spectrum (Fig. 2). Figure 2a, b demonstrates that only subtle changes in the impedance magnitudes and spectra were observed upon the conjugation reaction of SA and the subsequent immobilization of the S-proteins. The observed subtle impedance change resulting from the immobilization of the recognition layer can be associated with the relatively lower sensitivity of capacitive-based detection compared to Faradaic-based detection^57–67,75^.

### Electrical equivalent circuit model of a capacitive immunosensor

**Fig. 3:**
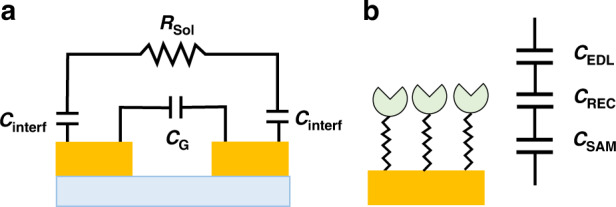
Model equivalent circuit of biosensor and the interfacial capacitance representing each layer formed on surface functionalization and probe immobilization. Schematic representation of **a** a simplified model equivalent circuit constituting a capacitive immunosensor and **b** interfacial capacitors formed by surface functionalization and S-protein immobilization of the IMA.

Figure 3a presents a simplified electrical model of our capacitive immunosensor, which uses a pair of adjacent gold electrodes in the IMA, depicting the electrical connections to the three essential circuit elements *C*_G_, *R*_sol_, and *C*_interf_, in which *C*_G_ is the geometric or parasitic capacitor, *C*_interf_ is the interfacial capacitor arising from the surface functionalization and spike protein immobilization on the surfaces of the electrodes and the gaps between the electrodes of the IMA, and *R*_sol_ is the sample solution resistance. The three circuit elements are connected in two parallel branches, in which one branch is formed by a series combination of the solution resistance (*R*_sol_) and the interfacial capacitance (*C*_interf_), and the other branch is represented by the geometric or parasitic capacitance (*C*_G_); this branch is contributed to by the solvent medium and the layers formed by surface functionalization and spike protein immobilization of the gap region of the glass substrate. Figure 3b depicts a schematic representation of three serially connected capacitors that contribute to the interfacial capacitance (*C*_interf_) of the capacitive biosensor^57–67^. According to the model, the interfacial capacitance of our capacitive immunosensor is expressed as follows:

1 $$\frac{1}{{C_{{\rm{interf}}}}} = \frac{1}{{C_{{\rm{SAM}}}}} + \frac{1}{{C_{{\rm{REC}}}}} + \frac{1}{{C_{{\rm{EDL}}}}}$$

where *C*_SAM_ is the capacitance due to the SAM layers, *C*_REC_ is the capacitance due to contributions from the layers formed by the immobilized spike proteins and the target antibodies, and *C*_EDL_ is the capacitance due to the electrical double layer in the solution, which in turn is a series combination of the Stern layer (*C*_SL_) and diffuse layer (*C*_Diff_).

### Label-free non-Faradaic EIS detection of antibodies

The detection signal in an EIS-based biosensor can arise from either Faradaic or non-Faradaic processes, which occur at the electrodes due to selective antigen-antibody binding in the recognition layer. In Faradaic-based biosensors, the detection signal arises from the interfacial charge-transfer reaction on the IMA due to the oxidation‒reduction reaction of the redox reagents present in the system. These reagents must be added to the system and require voltages higher than the redox potential of the redox reagents to be applied. In contrast, the detection signal in non-Faradaic-based biosensors arises from the change in capacitance and resistance in the interfacial layer due to selective antigen-antibody binding in the recognition layer at the electrode-solution interface of the IMA. Hence, non-Faradaic is label-free and requires nonperturbing low voltages, which are suitable for POC applications. According to the Gouy Chapman Stern Model, the EDL is a series combination of the Stern layer, which is composed of a compact layer of immobile ions that are strongly adsorbed to the electrode surface and the diffuse layer in which the ions are mobile. The total EDL capacitance, which consists of the Stern layer (*C*_SL_) and diffuse layer (*C*_Diff_) capacitances in series, is dominated by the latter (1/*C*_EDL_ = 1/*C*_SL_ + 1/*C*_Diff_) when the electrolyte concentration and surface potential are large.

When the electrodes and the gaps between the electrodes in the IMA are functionalized with MUOH and APTES, respectively, as shown in Fig. 1, the Stern and diffuse layers are pushed away from the electrode surface by the formation of a self-assembled monolayer (SAM). Further functionalization of the SAM and subsequent spike protein immobilization led to the formation of the recognition layer, which allowed the sensor to selectively bind and detect the COVID-19 antibody.

It is well known that capacitive biosensors are less sensitive than Faradaic biosensors. An ingenious approach to enhancing the sensitivity of capacitive biosensors is to decrease the ionic strength of the solution used in the EIS measurements. The dramatic effect of ionic strength on capacitive immunosensor sensitivity and LOD has been reported in the literature^76^. Quantitatively, for a bare gold electrode, the EDL capacitance (1/*C*_EDL_ = 1/*C*_SL_ + 1/*C*_Diff_) formed in a solution can be considered as a series combination of capacitances formed by Stern and the diffuse layer. The Stern layer encompasses the inner and outer Helmholtz planes with capacitances on the order of 50 μF/cm^2 ^^57–67^. The diffuse layer thickness is defined by the Debye length, and its capacitance (*C*_Diff_) can be tuned by the ionic strength of the medium. Thus, a capacitive immunosensor sensor can be represented by a series combination of *C*_SAM_, *C*_REC_ and *C*_Diff_. Thus, a dramatic increase in the sensitivity of a capacitive immunosensor can be achieved by tuning the value of *C*_Diff_ to a value comparable to that of *C*_REC_ by simple dilution, decreasing the ionic strength of the medium. The thickness of the diffuse layer corresponds to the Debye length, which in turn defines the distance over which the applied electric field is screened by the ions in the solution. For sensitive detection of the target antibodies, which bind to the surface of the recognition layer and are exposed to the medium, the antibodies should be within the effective range of the applied electric field. The electric field decays exponentially with increasing distance from the electrode, and most of the field decays in a region that is determined by the size of the EDL, which at ambient temperature can be approximated by the Debye length^77^, *λ* = 0.304/√*I*, where *λ* is in nm and *I* is the ionic strength of the medium in M (mol/l). For example, 1x PBS and 0.001x PBS have *λ* values of 0.7 nm and 23.2 nm, respectively. To gain insight into the effect of ionic strength on the device sensitivity, we studied the device performance using MUOH- and APTES-modified IMA, as depicted in Fig. 1b, as an example to detect anti-S-protein antibodies (IgG, IgM and IGA) by measuring the impedances in solutions containing 1x PBS and 0.001x PBS.

**Scheme 1:**
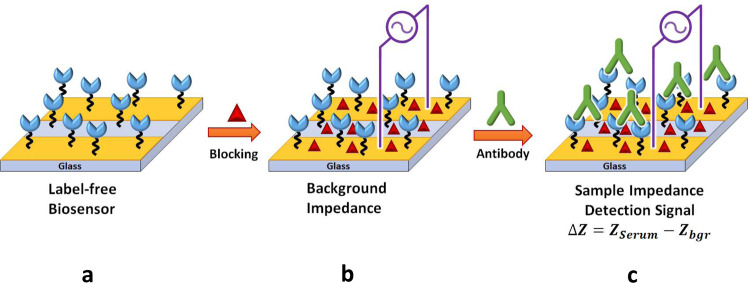
Schematic rendering of the label-free biosensor non-Faradaic EIS detection of the selective binding of antibodies to the trimeric spike proteins immobilized on the recognition layer of the impedimetric immunosensor. Ready-to-use biosensor **a** obtained after surface functionalization and trimeric spike protein immobilization. Non-Faradaic EIS detection was accomplished first by measuring the impedance (*Z*_bgr_) after blocking **b** with milk solution. In the second step, the impedance response (*Z*_Serum_) due to the affinity-specific captured antibodies **c** was measured. The detection signal (ΔZ) was obtained from the difference between these two measurements, i.e., Δ*Z* = *Z*_Serum_ – *Z*_bgr_. The sensors were thoroughly rinsed with 0.001× PBS pH 6.5 buffer after blocking (**b**) and after serum sample treatments (**c**) to minimize unintended contamination from the 1x PBS solution. All EIS measurements were conducted in 0.001× PBS solution at pH 6.5.

Scheme 1 presents the strategy adopted for the label-free biosensor assay of anti-SARS-CoV-2 immunoglobulin in serum samples using non-Faradaic EIS detection. The IMA-based biosensor was fabricated by sequential functionalization with APTES, MUA, and SA, followed by covalent immobilization of trimeric spike protein using EDC/NHS chemistry. To analyze the serum samples, the ready-to-use biosensor (Scheme 1a) was incubated in 1.5% milk in 1x PBS 0.05% TW20 pH 7.4 solution (Scheme 1b). After washing sequentially with 1x PBS 0.05% TW20 pH 7.4 solution and 0.001x PBS pH 6.5 solution, the background EIS response (*Z*_bgr_) of the sensor was measured in 0.001x PBS pH 6.5 solution; then, the sensor was incubated with the serum sample containing the target anti-SARS-CoV-2 immunoglobulin (Scheme 1c). After washing sequentially with 1x PBS, 0.05% TW20 pH 7.4 solution and 0.001x PBS pH 6.5 solution, the sample EIS response (*Z*_Serum_) arising from affinity-specific capture of the antibodies by the immobilized trimeric spike probes on the sensor was measured in 0.001x PBS pH 6.5 solution. The detection signal for the presence of the antibodies in the serum samples was evaluated from the difference, Δ*Z* = *Z*_Serum_ − *Z*_bgr_, where *Z*_bgr_ and *Z*_Serum_ are impedances of the IMA sensor measured before (but after incubation with 1.5% milk) and after incubation with COVID-19-positive serum, COVID-19-negative serum, or blank sample.

**Fig. 4:**
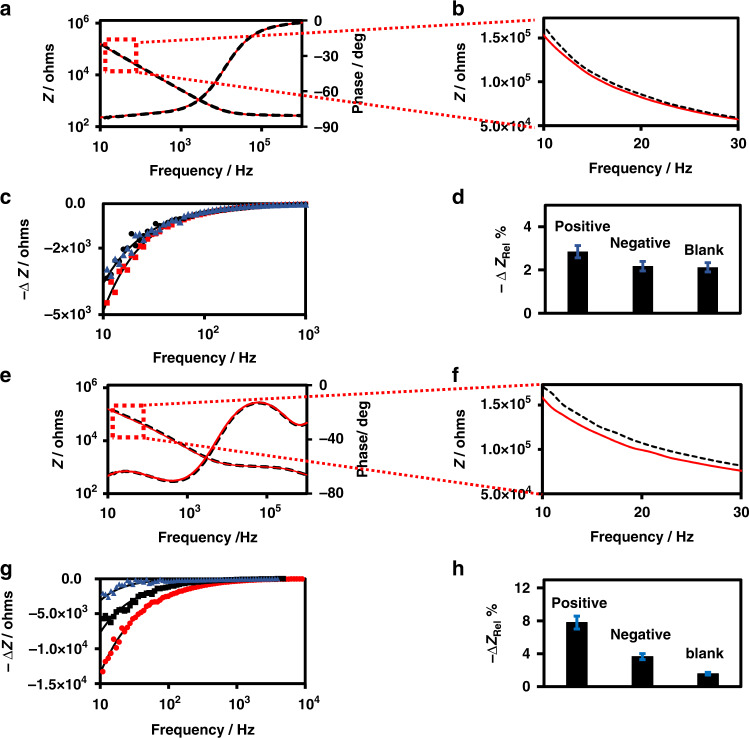
Dependance of the biosensor sensitivity on the ionic strength of the sensing medium for Non-Faradaic EIS detection. Non-Faradaic EIS detection of selective binding of antibodies to the S-proteins immobilized on the recognition layer of the impedimetric immunosensor comparing the effect of PBS concentration on the device sensitivity using MUOH- and APTES-modified IMA. (**a**, **e**)Bode plot of experimental EIS spectra before (black dash line) and after (redline) incubation with serum sample, (**b**, **f**) EIS spectra plotted in expanded scale to show the impedance change, (**c**, **g**) plot of Δ*Z* (Δ*Z* = *Z*_Serum_– *Z*_bgr_) versus frequency, () COVID-19 positive serum, () COVID-19 negative serum, () 1% milk blank samples, solid lines are drawn to guide the eyes, and (**d**, **h**) plot of the detection signal Δ*Z*_Rel_ % (Δ*Z*_Rel_ % = Δ*Z*/*Z*_bgr_ × 100).Plots (**a**–**d**) and (**e**–**h**) correspond to EIS measurements made in 1× PBS or 0.001× PBS solution at pH 6.5, respectively.

Figure 4a shows typical Bode plots for the EIS spectra of a typical IMA in 1x PBS solution, and the measurements were recorded before and after 2 h of incubation in COVID-19-positive serum. As seen in the expanded scale (Fig. 4b), the specific binding of the anti-spike antibodies (IgG, IgM, and IgA) to the S-proteins in the recognition layer of the IMA led to a decrease in impedance in the low frequency region of the EIS spectrum. To visualize the impedance change (Δ*Z*) resulting from the binding of antibodies to the S-proteins, difference spectra (Δ*Z* vs. frequency) obtained for COVID-19-positive, COVID-19-negative, and blank serum samples are presented in Fig. 4c, in which the control blank corresponds to a sample that was incubated with 1% fat-free milk in 1x PBS-0.05% TW20 at pH 7.4. The difference spectrum for each sample is plotted as Δ*Z* vs. frequency, where Δ*Z* = *Z*_Serum_ − *Z*_bgr_, where *Z*_bgr_ and *Z*_Serum_ are impedances of the IMA measured before and after incubation with COVID-19-positive serum, COVID-19-negative serum, or blank sample. Clearly, the magnitude of Δ*Z* increases rapidly with decreasing frequency in the low frequency region of the spectrum, whereas at frequencies above 1 kHz, the magnitude of Δ*Z* decreases to a negligibly small value. The observed functional dependence of Δ*Z* on frequency is as expected for a sensor based on capacitive detection. Figure 4d shows a bar plot of the detection signal (Δ*Z*_Rel_% = Δ*Z*/*Z*_bgr_ × 100) measured at 13.2 Hz in triplicates obtained for the COVID-19-positive, COVID-19-negative, and blank samples. The detection signals Δ*Z*_Rel_% observed were −2.9 ± 0.3, −2.2 ± 0.2, and −2.1 ± 0.2 for the COVID-19-positive serum, COVID-19-negative serum, and blank samples, respectively, and showed only a relatively small difference of 1.3-fold between the positive and negative serum samples. This result shows that the device exhibited a relatively low sensitivity when the impedance measurements were performed in 1x PBS solutions.

Figure 4e displays typical Bode plots of the EIS spectra of an IMA in 0.001x PBS solution recorded before and after 2 h of incubation in COVID-19-positive serum. Figure 4f shows that specific binding of the total immunoglobulin (IgG, IgM, and IgA) to the S-proteins in the recognition layer of the IMA led to a decrease in impedance in the low frequency region of the EIS spectrum, which was expected for a capacitive biosensor. Figure 4g shows the plots of Δ*Z* vs. frequency for COVID-19-positive, COVID-19-negative, and blank serum samples. The result shows that the magnitudes of Δ*Z* in the low frequency region measured in 0.001x PBS solution are much larger than those of the corresponding values in 1x PBS solution. The detection signals expressed in % relative values, Δ*Z*_Rel_%, are −7.8 ± 0.7, −3.7 ± 0.4, and −1.6 ± 0.2 for positive, negative, and blank samples, respectively. Hence, a significantly large difference (2.1-fold) between the COVID-19-positive and COVID-19-negative serum samples was observed compared to the value (1.3-fold) observed in 1x PBS solution. Clearly, the results presented here demonstrated that the sensitivity of the impedimetric immunosensor can be significantly improved by tuning the ionic strength of the media, which agrees with the theoretically predicted distance dependence of the electric field and quantitatively by tuning *C*_Diff_^76,77^. We also assessed the performance of the biosensor under different conditions, such as a shorter incubation time, serum samples from different patients, and using MUA instead of MUOH as the SAM layer. Serum samples from different patients produced varying values of Δ*Z*_Rel_% for positive COVID-19 cases, which was consistent with the known variation in total immunoglobulin titer with the patient and the time when the serum samples were collected^35–48^. In addition, the background impedance signal originating from the serum sample was expected to vary with the patient, as the serum samples of different patients were expected to contain many endogenous antibodies of varying concentrations. For example, when two sets of different COVID-19 positive and negative patient samples were tested for the same incubation time of 1 h, the Δ*Z*_Rel_% of the first set yielded −1.6 and −0.3 for the COVID-19 positive and the negative patients, respectively, whereas corresponding values for the second set yielded −3.1 and −2.3, indicating that the Δ*Z*_Rel_% positive/negative ratios varied from 6.0 to 1.3. We also expected that using MUA instead of MUOH, as depicted in Fig. 1c, could enhance the device performance; this enhancement resulted from the increased immobilization of S-proteins on the gold surface of IMA.

**Fig. 5:**
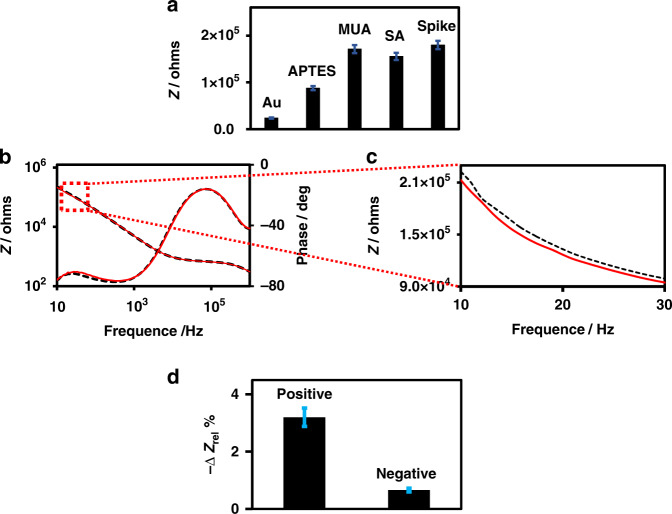
Effect of spike protein coated gold electrode on the biosensor sensitivity. **a** Impedance magnitudes recorded after different sequential stages of surface functionalization with APTES, MUA, SA, and S-protein immobilization. **b** As depicted schematically in Figure 1, Bode plot of the experimental EIS spectra before (black dashed line) and after (redline) incubation with the serum sample. **c** Magnified EIS spectra plotted to show the impedance change. **d** Plot of the detection signal Δ*Z*_Rel_ % (Δ*Z*_Rel_ % = Δ*Z*/*Z*_bgr_ × 100). All EIS measurements were performed in 0.001× PBS.

Figure 5a shows the impedance magnitudes (*Z*) at 13.2 Hz after the IMA underwent different stages of surface functionalization with APTES, MUA, SA and subsequently after S-protein immobilization, as depicted schematically in Fig. 1. A dramatic change in impedance was observed after the gap and Au surfaces of the IMA underwent APTES and MUA functionalization, respectively. Similar to the trend presented in Fig. 2, S-protein immobilization led to a relatively small and subtle change in impedance. This sensor can be expected to exhibit higher sensitivity, as both the gap and Au surfaces of the IMA are coated by the probe, as depicted in Fig. 1c. The test results for a set of patient serum samples, as shown in Fig. 5d, yielded Δ*Z*_Rel_% values of −3.2 and −0.7, corresponding to a positive/negative ratio of 4.9, indicating enhanced performance. Hence, our impedimetric capacitive immunosensor exhibits a demonstrated capability to qualitatively detect the presence or absence of COVID-19 antibodies in human serum samples. Based on the limited number of human serum samples we analyzed, which included three COVID-19-positive and three COVID-19-negative samples, the test results of our impedimetric capacitive immunosensor are in good agreement with the results provided by the Alberta Health service (AHS) in which two different assays, Abbott ARCHITECT SARS-CoV-2 IgG (Nucleocapsid) and DiaSorin LIAISON SARS-CoV-2 IgG (Spike), were used; thus, the results demonstrate the excellent sensitivity and specificity of the device.

### Model electrical equivalent circuit analysis

Based on the simplified model presented in Fig. 3a, the total impedance (*Z*) of the system as a function of the applied frequency (*f*) in an EIS spectrum is contributed to by three circuit elements, which are connected in two parallel branches; one branch is formed by a series combination of the solution resistance (*R*_sol_) and the interfacial capacitance (*C*_interf_) and the other branch is represented by the geometric or parasitic capacitance (*C*_G_) expressed by the following equation^57–67^:

2 $$\frac{1}{Z} = \frac{1}{{\sqrt {R_{{\rm{sol}}}^2 + \frac{1}{{\left( {\pi fC_{{\rm{interf}}}} \right)^2}}} }} + 2\pi fC_{\rm{G}}$$

By examining Eq. (2), it was found that at low frequencies, the impedance is determined by the branch that is formed by a series combination of the solution resistance (*R*_sol_) and the interfacial capacitance (*C*_interf_); this is because the current cannot pass through the geometric capacitor *C*_G_, and thus, the circuit is inactive or open. As the impedance due to *R*_sol_ is small compared to *C*_interf_, *C*_interf_ becomes the dominant contributor to the impedance in the low frequency region. To account for a variety of nonideal conditions present in the sensing system, such as surface irregularities, chemical heterogeneities, and uneven ion adsorption onto the electrode surface, the double layer capacitance is better represented by the constant phase element (CPE), and the impedance (*Z*_CPE_) is given by *Z*_CPE_ = 1/(*jω*)^*n*^*Q*, where *j* is the imaginary unit, *ω* = 2*πf*, and *Q* is equivalent to the capacitance of a perfect capacitor, *Q* = (*πf*)^1−*n*^ *C*_interf_. The coefficient *n* of CPE varies between 0 and 1. When *n* = 1, *Q* becomes a perfect capacitor *C*_interf_.

**Fig. 6:**
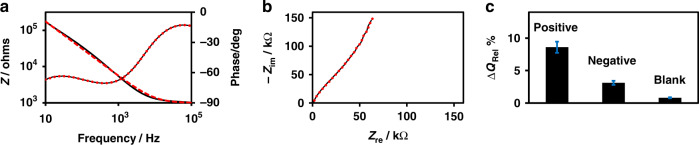
Comparison of the experimental and simulated EIS data points obtained from the model equivalent circuit. Experimental (black line) and simulated (red dashed line) EIS spectra for detecting the selective binding of antibodies to the S-proteins immobilized on IMA modified with MUOH and APTES plotted in **a** Bode and **b** Nyquist representations and **c** plot of the capacitance change Δ*Q*_Rel_% obtained from the simulation.

A key step to better understand and improve the performance of the capacitive biosensor is to build model equivalents of electrical circuits that represent the system and evaluate the contributions from all the components. To understand and quantify the capacitance change resulting from the binding of total immunoglobulin onto the recognition layer coated with S-protein in the device, we simulated the experimental data presented in Fig. 4e–h with the model equivalent circuit presented in Fig. 3a, b using Eq. (2). Table 1 lists the equivalent circuit parameters *n*, *Q*, and *R*_sol_ that were obtained by fitting the EIS experimental data. Figure 6a–c shows the Bode magnitude and phase plot and Nyquist plot of the experimental impedance magnitude of a typical COVID-19-positive serum sample, that is, the EIS data presented in Fig. 4e, and the corresponding simulated curve obtained using the parameters listed in Table 1. The agreement between the experimental and fitted curves at frequencies below 100 kHz is remarkable, given the simplicity of the model equivalent electrical circuits used in the simulation. The samples, positive, negative, and control blank and the corresponding background EIS spectra can be simulated with the same medium resistance of 1 kΩ as expected. A significant deviation in the parameter *n* from unity indicates the nonideal capacitor characteristics of the IMAs and suggests the areas that need further improvements. Interestingly, Table 1 shows that the binding of total immunoglobulin to the S-proteins results in an increase in capacitance of the system, e.g., the capacitance increases from 430 to 467 nF when the antibodies bind. This increase is also reflected in the EIS spectrum shown in Fig. 4f, in which antibody binding led to a decrease in the impedance, and from the negative values for Δ*Z*_Rel_% in Fig. 4 and Table 1. Similar results were observed in our recent work on capacitive immunosensors for canola pathogens^78^. Figure 6c shows the bar plot for Δ*Q*_Rel_%, which clearly indicates the applicability of the impedimetric immunosensor presented here for the detection of antibodies against COVID-19 in serum samples.

**Table 1:** Equivalent circuit parameters *n*, *Q*, and *R*_sol_ were obtained by fitting the experimental data with the equivalent circuit depicted in Fig. 3.

Sample	*R*_sol_/kΩ	*n*	*Q*/nF	*Q*/nF	Δ*Q*_Rel_%	*Z*/kΩ	*Z*/kΩ	Δ*Z*_Rel_%
			Background	Sample		Background	Sample	
Positive	1.0	0.75	430.0	467.0	8.6	143.0	131.0	−7.8
Negative	1.0	0.75	385.0	397.0	3.1	159.0	153.0	−3.7
Blank	1.0	0.81	247.0	249.0	0.8	167.0	165.0	−1.6

The experimental impedance data plotted in Fig. 4h are included for comparison. Detection signals presented as Δ*Z*_Rel_% and Δ*Q*_Rel_% were evaluated from the experimental impedance magnitudes and simulated capacitances, respectively. All values are average values of three different samples and have an estimated RSD of ~10%.

An important problem that needs to be addressed for immunosensors is the specificity of the recognition element to the target antibodies and the potential cross reactivity with other closely related antibodies. Detailed tests on the cross reactivity of the S-protein used as the recognition layer in our biosensor against antibodies of other closely related pathogens are ongoing. It is important to note that the samples tested in this work are raw serum samples that contain many endogenous antibodies and biomolecules. The results of the serum samples tested using the biosensor developed in this work agree with the tests performed with gold standard ELISA and CLIA methods. Hence, the capacitive immunosensor presented here can be used for label-free detection of COVID-19 antibodies in human serum.

Recently, several papers reported the development of electrochemical biosensors for the rapid detection of SARS-CoV-2 antibodies^68–71^. These papers claimed to have achieved rapid detection times of a few minutes to even a few seconds. For the biosensor reported here, the tests performed thus far showed that the sensor can detect SARS-CoV-2 antibodies in serum samples in less than 1 h. This is a long time compared to a few seconds but is significantly better than the time needed for typical ELISA tests^49–56^. We are continuing to optimize the assay procedure for the biosensor and expect to achieve a significantly shorter assay time in the future.

### Standard curve and assay performance comparison

**Fig. 7:**
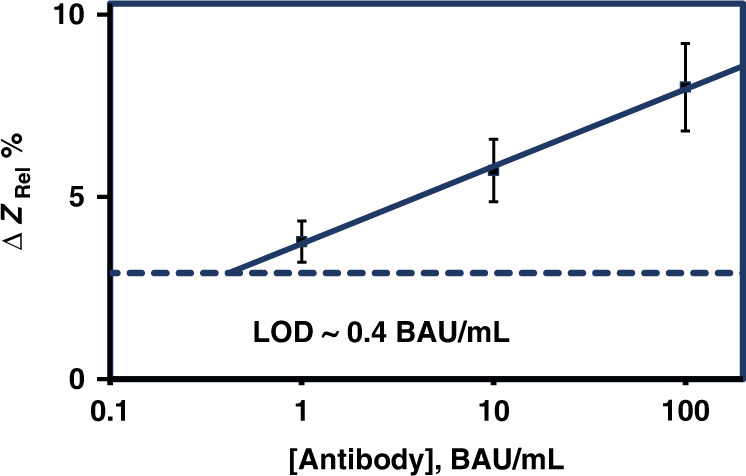
Standard calibration curve for the detection of anti-SARS-CoV-2 antibodies obtained using label-free non-Faradaic EIS biosensor and comparison of its performance with other biosensors. Standard calibration curve for the detection of anti-SARS-CoV-2 immunoglobulin (IgG, IgA, IgM), which were experimentally determined in duplicate (*n* = 2) using different concentrations of WHO IS (NIBSC 20/136) antibody solutions. Linear fit (solid line) to the experimental data points (filled squares) yielded a straight line, Δ*Z*_Rel_% = 2.1178 log [BAU/mL] + 3.7173 with R^2^ = 0.9979, indicating that the highest concentration used, i.e., 100 BAU/mL still lied within the linear region of the impedance response (Δ*Z*_Rel_ % = Δ*Z*/*Z*_bgr_ × 100) to antibody concentration. The dotted line represents the LOD determined fromthe impedance response Δ*Z*_Rel_% of the negative serum sample with no anti-SARS-CoV-2 immunoglobulin. For ease of representation, the bindingantibody unit BAU/mL is used to express the concentration of all immunoglobulins (IgG, IgA, IgM), and according to WHO/NIBSC both (IU/mL and BAU/mL) units are numerically identical. All EIS measurements were conducted in 0.001× PBS at pH 6.5.

Two configurations of the devices are presented in Fig. 1 which differ by the nature and region of surface functionalization and probe immobilization. They are designed to mitigate the effects of nonspecific binding and enhanced capacitive response when the target antibodies are captured on the IMA sensor surface. The device based on the SAMs of APTES and MUA which functionalized both gaps as well as the gold digits is expected to enhance the device performance due to increased coverage of the sensor by the probe spike proteins, and this device configuration was chosen to determine the standard curve. To facilitate the evaluation of the device and directly compare its performance with the established commercially available immunoassays and other devices reported in the literature, we used the World Health Organization International Standard (WHO IS) for anti-SARS-CoV-2 immunoglobulin (NIBSC 20/136)^79,80^. Figure 7 shows the plot of the experimental impedance response Δ*Z*_Rel_% vs. antibody concentration (BAU/ml). The trimeric spike protein (S1, S2, and RBD) recognition element used in the sensor device detects the total antibody, anti-SARS-CoV-2 immunoglobulin (IgG, IgA, IgM). Therefore, for ease of representation, we uniformly used binding antibody units BAU/ml to express the concentration of all immunoglobulins (IgG, IgA, IgM), and this did not introduce error because, according to WHO/NIBSC, both (IU/ml and BAU/ml) units are numerically identical. A linear fit to the experimental data yielded a straight line with an *R*^2^ value of 99%. The limit of detection (LOD) based on negative serum samples was 0.4 BAU/ml, and a slightly lower value was obtained from the milk as background and from estimates based on the standard deviation of the background.

Table 2 lists the limit of detection (LOD), upper detection limit (UDL), target antibody class detected, recognition antigens used as the probe, and the type of signal transduction used by the devices to detect the antibody present in the serum samples. Interestingly, an LOD of 0.4 BAU/ml was determined for our device and is similar to the corresponding LODs reported for all the validated and established commercial assays, which are within a range of 0.41–4.81 BAU/ml^81^. Rashed et al. reported that the lowest concentration measured was 0.27 BAU/ml, which is close to the corresponding values for the established commercial assays. However, the LODs reported by Ali et al. and Yakoh et al. are several orders of magnitude lower than the corresponding values for the established commercial assays. A valid question is how and why these values are so different. A reason for the difference may arise from the affinity and specificity of the commercially purchased antibodies to the epitopes of the recognition antigens used in these devices. The question can also be considered from the viewpoint that the initial WHO study reported very large (several orders of magnitude) interlaboratory variations for the ELISA.

**Table 2:** Comparison of the performance of recently developed electrochemical-based biosensors and established and commercially available assays for anti-SARS-CoV-2 immunoglobulins.

Target antibody class	Recognition antigen	Signal transduction	LOD (BAU/ml)^a^	UDL (BAU/ml)^a^	Reference
Total Ab (IgG, IgA, IgM)	Trimeric spike	EIS	0.4	>100	This work
IgG	RBD	Transient EIS	0.27^b^	27.0	^68^
IgG	S1	Faradaic EIS	9.2E−07	3.3	^69^
IgG	RBD	Faradaic EIS	6.8E−06	0.4	^69^
IgM	RBD	Faradaic SWV	1.4E−02	14.20	^70^
IgG	RBD	Faradaic SWV	2.7E−03	2.70	^70^
IgG	Trimeric spike	CLIA (DiaSorin)	4.81	2080	^81^
IgG	S1/S2	CLIA (DiaSorin)	3.8	400	^81^
IgG	RBD	CMIA (Abbott)	2.98	5680	^81^
Total Ab (IgG, IgA, IgM)	RBD	ECLIA (Roche)	0.41	257.2	^81^
IgG	RBD	ELISA (Euroimmun)	3.2	384	^81^

^a^Limit of detection (LOD), upper detection limit (UDL), Abbott SARS-CoV-2 IgG II Quant chemiluminescent microparticle immunoassay (CMIA), Roche Elecsys® Anti-SARS-CoV-2 electrochemiluminescence immunoassay (ECLIA), Euroimmun anti-SARS-CoV-2 QuantiVac enzyme-linked immunosorbent assay (ELISA), DiaSorin LIAISON® SARS-CoV-2 S1/S2 IgG immunochemiluminescent assay; indirect immunoassay. Chemiluminescence detection (CLIA).

^b^The lowest IgG concentration analyzed and not the experimental LOD. Equation^87^ relating WHO IS antibody concentration to nanogram/ml (ng/ml) is given by BAU/ml = ng/ml × Conversion Factor, and the Conversion Factor values^87^ are 0.0027, 0.0022, and 0.0142 for RBD specific to IgG, IgG specific to spike S1, and RBD specific to IgM, respectively.

## Conclusions

In this work, we presented the development of a label-free impedimetric capacitive immunosensor and demonstrated that the proof-of-concept device can selectively detect the total COVID-19-positive antibodies present in human serum samples; this is achieved by using the SARS-CoV-2 spike protein as the probe. Hence, the sensor can clearly differentiate COVID-19-positive from COVID-19-negative human serum samples with excellent sensitivity and specificity. We demonstrated that a significant increase in the sensitivity of a capacitive immunosensor can be achieved by decreasing the ionic strength of the sample solution. The non-Faradaic impedimetric detection presented here uses low nonperturbing voltages and requires no labeling or addition of sensing reagents, and unlike ELISA, the method does not require enzyme-labeled secondary antibodies, substrates, or the associated incubation times. Hence, a label-free impedimetric immunosensor inherently requires a significantly shorter incubation time than that of ELISA, and we expect a rapid diagnostic test to be achievable. In addition, the device can be readily used for rapid antigen tests to detect active SARS-CoV-2 infection by interchanging the immobilized S-proteins in the recognition layer of the biosensor with antibodies. The standard calibration curve of our biosensor, with covalently immobilized trimeric spike protein as the recognition element, which was determined using WHO IS anti-SARS-CoV-2 immunoglobulins, yielded an LOD of 0.4 BAU/ml; this value is comparable to the corresponding values reported for the validated and established commercial assays.

## Materials and methods

### Preparation and purification of the S-protein

The SARS-CoV-2 spike protein (GenBank: QHD43416.1) was codon optimized and modified to remove the transmembrane domain region. It was synthesized on a pABbee™-FH plasmid by GenScript (USA). Recombinant baculovirus was generated by transfecting the spike plasmids into Sf9 cells (Expression Systems) using the ProFold™-ER1 system (AB vector). Clones were screened following plaque purification, and master seed stocks were prepared, characterized for identity, and used to prepare working virus stocks. The working stocks were subcultured on Sf9 cells at 27 °C with shaking at 130 rpm for 120 h and were subsequently used to infect suspension cultures of TNI cells (Expression Systems) at an MOI of 5–10. The infected TNI cells were incubated at 27 °C with shaking at 130 rpm for 72 h for protein expression. The spike protein was purified by nickel-nitrilotriacetic acid (Ni-NTA) (Qiagen, Toronto, Canada), and the final product was confirmed to be functional by evaluation of the antigen using an in-house developed ELISA with positive and negative SARS-CoV-2 sera.

### Human serum samples

Blood samples from patients who had been tested and confirmed to be COVID-19-positive and COVID-19-negative by reverse transcription–polymerase chain reaction (RT–PCR) were collected in Becton Dickinson SST tubes (containing silica clot activator) and centrifuged according to the manufacturer’s instructions to separate the serum. Human serum samples were categorized and verified as either COVID antibody-positive or COVID antibody-negative by the AHS using the following different assays: Abbott ARCHITECT SARS-CoV-2 IgG (Nucleocapsid) and DiaSorin LIAISON SARS-CoV-2 IgG (Spike). This work was conducted in accordance with approval and guidance that was provided by the research ethics boards at the University of Calgary (Study ID REB20-0516) and the University of Alberta (Study IDs Pro00099818 and Pro00109215). All study participants were above the age of 18 and provided full informed consent to participate, as per guidance by research ethics boards.

The WHO IS for Anti-SARS-CoV-2 Immunoglobulin was purchased from the National Institute for Biological Standards and Control (NIBSC, Hertfordshire, UK) and was provided as ampoules with 250 µl per ampoule equivalent to 1000 binding antibody units (BAU) or international units (IU) per milliliter. A negative plasma base matrix was used to prepare samples with different concentrations.

### Materials and reagents

Borofloat glass wafers were purchased from Swift Glass Co., Inc. Elmira Heights NY. 1-Mercapto-11-undecanoic acid 97% (MUA), 11-mercapto-1-undecanol 99% (MUOH), 3-aminopropyltriethoxysilane 99% (APTES) N-(3-(dimethylamino)propyl)-N′-ethylcarbodiimide hydrochloride (EDC), N-hydroxysuccinimide 98% (NHS), ethyl acetate 99.8% (EA), succinic anhydride 99% (SA), ethanol 100% (EtOH), acetone (AC), isopropanol (IPA), disodium hydrogen phosphate, monosodium hydrogen phosphate, and bovine serum albumin 98% (BSA) were purchased from Sigma-Aldrich Canada Co. (Oakville, Ontario) and were used without further purification. Commercially available fat-free milk was used without further purification. Ultrapure water (18.2 MΩ cm at 25 °C) was obtained from Millipore equipment (Milli-Q water) for sample preparation and washing.

### Instruments

**Fig. 8:**
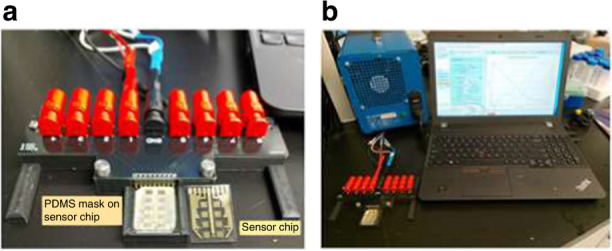
Instruments and accessories for experimental EIS data acquisition. Custom built electrical contact pad, sensor chip, and sensor chip with a PDMS mask that contained eight matching wells (**a**). Experimental setup consisting of a Biologic SP-200 potentiostat and the associated computer control system for EIS measurements (**b**) of the affinity-bound target analytes on the sensor chip.

EIS measurements were obtained with a potentiostat/galvanostat SP-200 controlled by EC lab software from BioLogic Science Instruments Inc. (Knoxville, Tennessee). A custom-built electrical contact pad with a connector adapter was used to form electrical connections between SP-200 and the IMAs on the sensor chips similar to those reported earlier^78,82–85^. Sirus T2 Tabletop Reactive Ion Etcher, Trion Technology, Clearwater, Florida, was used for plasma cleaning. Figure 8 shows the setup of the EIS measurements in the laboratory, which consists of a custom-made electrical contact pad with pogo pins for soft contact with the IDE electrodes, a bare sensor chip with 8 IMA, a sensor chip mated with a PDMS mask with 8 matching wells, a BioLogic SP-200 potentiostat, and the associated computer system for data acquisition.

### Fabrication of the interdigitated microelectrode array

**Fig. 9:**
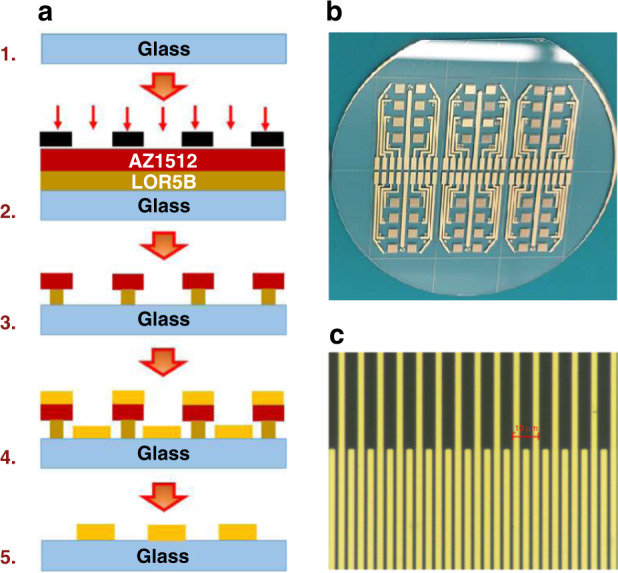
Schematic depiction of lift-off process flow for microfabrication of gold interdigitated microelectrode array. Standard lift-off microfabrication technique forfabrication of IMA (**a**): (1) borofloat glass wafer coated with hexamethyldisilazane, (2) UV light exposureof the bilayer coated wafer after spin coating and baking AZ1512 photoresist and LOR5B resist through a custom designed photomask using mask aligner, (3) after sequential development with AZ 1:1 and followed by MF 319 developers, (4) after metal deposition with 5 nm of a Cr adhesion layer and 60 nm gold, andfinally (5) after lift-off with remover-PG. Borofloat glass wafer withpatterned IMA electrode sensor chips obtained after lift-off (**b**), and image of interdigitated electrodes (**c**) with width, gap, andthickness of 4 µm, 2 µm, and 60 nm, respectively.

IMAs were fabricated using a standard lift-off microfabrication technique at the NanoFAB, Fabrication & Characterization Centre, University of Alberta, using a custom designed photomask and photolithography. Briefly, a clean borofloat glass wafer coated with a monolayer of hexamethyldisilazane was sequentially spin-coated with a bilayer composed of 500 nm of LOR-5B resist followed by 1.1 μm of AZ1512 photoresist. UV light exposure of the bilayer-coated wafer was performed through the custom designed photomask with the desired pattern using a mask aligner followed by development with AZ 1:1 and MF-319 developers, giving the wafers a pattern formed by the developed photoresists. Subsequently, a 5 nm Cr adhesion layer followed by 60 nm Au was deposited successively onto the wafer. The bilayer photoresists were “lifted-off”, leaving behind the patterned gold IMA on the wafer. Finally, the wafers were diced into chips, and each chip contained eight IMAs per chip. Figure 9 shows the schematic process flow for the fabrication of the sensor chips, wafer with the fabricated IMA patterns, and microscopic image of typical IMA gold electrodes with dimensions for the width, gap, and height of 4 µm, 2 µm, and 60 nm, respectively.

### Surface functionalization of the IMA chip and antigen immobilization

To functionalize the wafer, the glass chip was first cleaned by sonication in acetone, IPA, and DI water for 10 min each and exposed to oxygen plasma from Trion to burn off trace organics and activate them to form hydroxyl groups on the glass surface. The chip was then incubated for 3 h in 2.5% APTES solution in EtOH^82^, washed and sonicated with EtOH, and heated to 120 °C for 30 min on a hot plate to form an amine-terminated cross-linked SAM on the surface. After washing with EtOH, the chip was incubated in 0.7 mg/ml MUOH in ethanol overnight to form SAM of MUOH on the gold surface of the IMA. Subsequently, IMA with SAM of MUOH and APTES on the gold and glass surfaces was incubated with 11 mg/ml succinic anhydride (SA) in ethyl acetate for 3 h to produce a carboxylic acid group-functionalized glass surface^86^. To immobilize the S-proteins on the surface of IMA, a PDMA mask with 8 matching wells was used to form a well on each IMA^78,82–84^. Each well was incubated for 15 min in a 50 µl aliquot of solution containing 28.0 mg/ml NHS and 14.0 mg/ml EDC in 10 mM MES pH 5.5^78,83^. After washing, the wells were incubated for 1 h in a 50 μl aliquot of spike protein in 1x PBS (phosphate buffer saline) at pH 8. After washing with 1x PBS containing 0.05% TW20 at pH 7.4, the sensor chip was ready for capacitive immunoassay of antibodies against SARS-CoV-2.

### Capacitive immunoassay of SARS-CoV-2 antibodies

A polydimethylsiloxane (PDMS) mask with eight wells^78,82–84^ was useds to submerse the matching eight IMAs on the sensor chip that was filled with 50 μl of 10 μM PBS or 0.001x PBS at pH 7.4 for electrochemical impedance spectra (EIS) measurements. The EIS was recorded by applying ±10 mV sinusoidal excitation perturbation at 0 V DC and scanning the excitation frequency from 10 Hz to 1 MHz. The impedance at each frequency in the EIS spectrum is the average of five repeated measurements. To minimize nonspecific binding, each well in the sensor chip was incubated for 1 h in 50 μl of 2% fat-free milk in 1X PBS-0.05% TW20 at pH 7.4. After washing, the EIS spectrum of each well was recorded in 50 μl of 0.001x PBS at pH 7.4 and served as the background signal (*Z*_bgr_) of the detection. The wells were then incubated for 1–2 h in 50 μl aliquots of 1/10 dilution of serum samples in 1% fat-free milk containing 1X PBS-0.05% TW20 at pH 7.4. After washing, the EIS spectrum of each well was recorded in 50 μl of 0.001x PBS at pH 7.4 to serve as the sample signal (*Z*_Serum_) of the assay. The serum samples used in this analysis were tested by ELISA to be either positive or negative for the presence of anti-S-protein IgG antibodies. A blank sample containing 1% fat-free milk in 1X PBS-0.05% TW20 at pH 7.4 without serum sample was included in the test as the control sample. The detection signal was determined from the impedance change (Δ*Z*) observed at a frequency of 13.2 Hz, and the change arises from the binding of the total antibodies (IgG, IgM and IgA) to the S-proteins immobilized in the recognition layer of the IMA. The experimentally observed impedance change at 13.2 Hz can be presented as either difference Δ*Z* or relative change Δ*Z*_Rel,_ where Δ*Z* = *Z*_Serum_ − *Z*_bgr_ and Δ*Z*_Rel_ = (*Z*_Serum_ − *Z*_bgr_)/*Z*_bgr_.

### Statistical analysis

Data are presented as original data or means with error bars that represent the standard deviation obtained from three replicate experiments (*n* = 3). The impedance at each frequency in the EIS spectrum is the average of five repeated measurements. Data simulations were performed with nonlinear least-squares analysis using Origin or Excel.

## Data Availability

The data files, including raw data used to support the findings in this study, can be obtained from the corresponding author upon reasonable request.
